# Contribution of Smoothened Receptor Signaling in GABAergic Neurotransmission and Chloride Homeostasis in the Developing Rodent Brain

**DOI:** 10.3389/fphys.2021.798066

**Published:** 2021-12-10

**Authors:** Mira Hamze, Igor Medina, Quentin Delmotte, Christophe Porcher

**Affiliations:** ^1^Aix-Marseille University, INSERM, INMED, Parc Scientifique de Luminy, Marseille, France; ^2^INSERM (Institut National de la Santé et de la Recherche Médicale) Unité, Parc Scientifique de Luminy, Marseille, France; ^3^INMED (Institut de Neurobiologie de la Méditerranée), Parc Scientifique de Luminy, Marseille, France

**Keywords:** GABA, KCC2 activity, Smo, chloride homeostasis, Shh

## Abstract

In the early stages of the central nervous system growth and development, *γ-aminobutyric acid* (GABA) plays an instructive trophic role for key events including neurogenesis, migration, synaptogenesis, and network formation. These actions are associated with increased concentration of chloride ions in immature neurons [(Cl^−^)_i_] that determines the depolarizing strength of ion currents mediated by GABA_A_ receptors, a ligand-gated Cl^−^ permeable ion channel. During neuron maturation the (Cl^−^)_i_ progressively decreases leading to weakening of GABA induced depolarization and enforcing GABA function as principal inhibitory neurotransmitter. A neuron restricted potassium-chloride co-transporter KCC2 is a key molecule governing Cl^−^ extrusion and determining the resting level of (Cl^−^)_i_ in developing and mature mammalian neurons. Among factors controlling the functioning of KCC2 and the maturation of inhibitory circuits, is Smoothened (Smo), the transducer in the receptor complex of the developmental protein Sonic Hedgehog (Shh). Too much or too little Shh-Smo action will have mirror effects on KCC2 stability at the neuron membrane, the GABA inhibitory strength, and ultimately on the newborn susceptibility to neurodevelopmental disorders. Both canonical and non-canonical Shh-Smo signal transduction pathways contribute to the regulation of KCC2 and GABAergic synaptic activity. In this review, we discuss the recent findings of the action of Shh-Smo signaling pathways on chloride ions homeostasis through the control of KCC2 membrane trafficking, and consequently on inhibitory neurotransmission and network activity during postnatal development.

## Introduction

**Figure 1:**
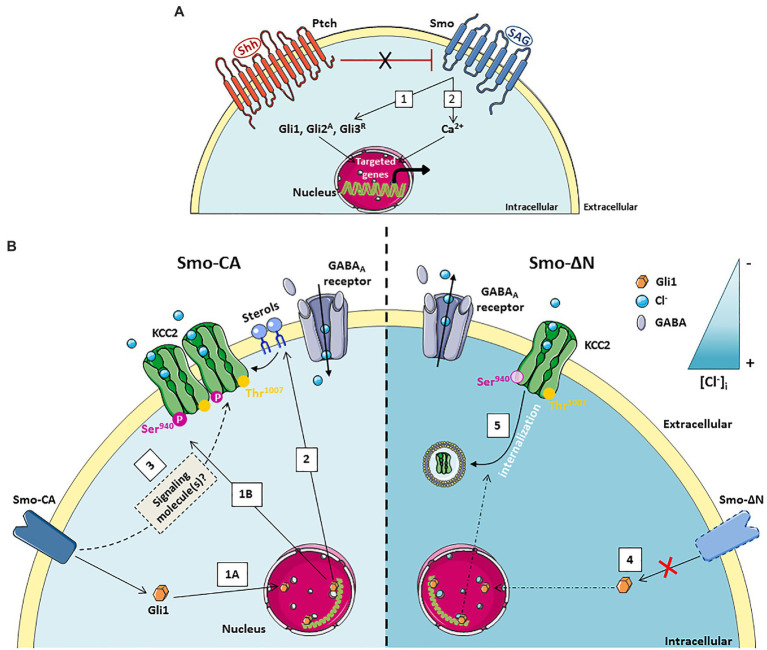
A schematic model of action of the Smoothened receptor signaling on GABAergic transmission in the immature hippocampus. (1) Activation of the non-canonical Smo signaling pathway by its ligand (SAG) leads to an intracellular calcium increase in the post-synaptic neuron which can be mediated either through calcium channels or by the release of intracellular stores from the endoplasmic reticulum. (2) The intracellular increase in calcium concentration triggers the exocytosis of BDNF. (3) BDNF binds to its receptor TrkB on neighboring synapses and (4) increases GABAergic synaptic transmission.

**Figure 2:**
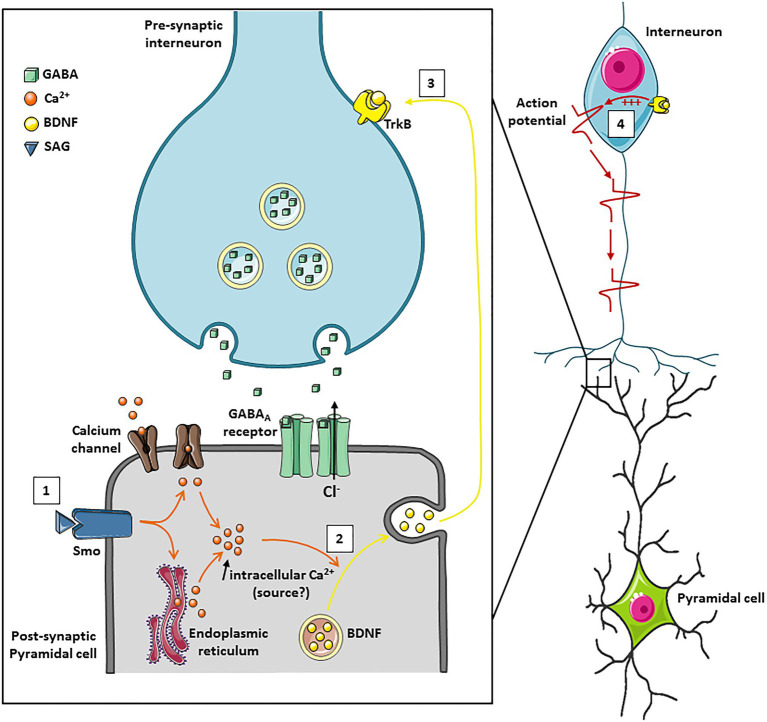
A schematic model of the Smoothened receptor signaling pathways controlling KCC2 activity. **(A)** In the absence of the ligand Shh, the Patched (Ptch) receptor maintains the Smoothened (Smo) receptor in an inactive state. Smo signaling can be activated either by the binding of Shh which inactivates Ptch and releases Smo or by the binding of its agonist SAG (Shh Signaling Agonist). Smo signaling can operate through: (1) canonical pathway which activates the transcription factors Gli allowing transcription of Gli target genes, (2) Non-canonical pathway that is Gli-independent but triggers transient calcium currents allowing transcription of targeted genes. **(B)** Left: (1a) Constitutive activity of the Smo-CA receptor promotes Gli1 downstream target genes expression. (1b) The Smo-Gli1 signaling pathways activate the phosphorylation of KCC2 at the residue Serine 940, increasing KCC2 function and stability at the cell surface, which renders GABAergic transmission less depolarizing. (2) The Smo/Gli1 signaling pathways may modify the synthesis/transport of sterols, impacting the composition of the plasma membrane and lipid rafts and the stability of KCC2 at the cell surface. (3) The Smo-Gli1 pathway may also regulate chloride ions homeostasis and KCC2 function through a second messenger. Right: (4) Dominant-negative activity of Smo-ΔN represses Gli1 expression. (5) Inhibition of Smo-Gli1 signaling pathway induces dephosphorylation of residue Serine 940 and KCC2 internalization.

Developing neuronal circuits generate primitive patterns of network activity, that are necessary to support more complex neuronal processes and future cognitive functions. These primitive activities require γ-aminobutyric acid (GABA) synaptic transmission and chloride flux through GABA_A_ ionotropic receptor channels (GABA_A_R). The inhibitory strength of GABA_A_R transmission is dependent on intracellular neuronal chloride concentration [(Cl^−^)_i_], that is relatively high (15–25 mM) in immature neurons and decreases progressively to 4–6 mM in mature neurons. This developmental change of (Cl^−^)_i_ is determined primarily by activity of two cation-chloride cotransporters sodium-potassium-chloride cotransporter type 1 (NKCC1) and potassium-chloride cotransporter type 2 (KCC2) (see reviews Medina et al., 2014; Virtanen et al., 2021). Several factors controlling the maturation of GABAergic transmission have been identified so far, including GABA itself, neurotrophic factors (Roussa et al., 2016; Porcher et al., 2018), pituitary neuropeptides (Tyzio et al., 2006; Leonzino et al., 2016; Spoljaric et al., 2017), peripheral metabolic and sex hormones (Galanopoulou and Moshé, 2003; Sawano et al., 2013; Dumon et al., 2018), and more recently the Sonic Hedgehog (Shh) peptide and its signal transducer Smoothened (Smo) receptor (Delmotte et al., 2020b). In this review, we discuss the recent achievements in Shh-Smo signaling contribution to developmental and functional maturation of GABAergic transmission (Figure 1) and chloride ion homeostasis in the postnatal rodent brain (Figure 2).

## Shh-Smo Signaling During Postnatal Brain Development

Shh is a secreted glycoprotein preserved through the evolution and mostly known for its morphogenetic role during early phases of central nervous system development such as neural cell proliferation, neural progenitor cell fate, or neuronal differentiation (Ruat et al., 2012, 2014; Briscoe and Thérond, 2013; Belgacem et al., 2016). In the postnatal rodent brain the Shh signaling pathway, composed by the receptors complex Patched (Ptch) and Smo, is involved in neurogenesis (Breunig et al., 2008; Shqirat et al., 2021), growth of presynaptic terminals (Mitchell et al., 2012), connectivity of corticofugal projection neurons (Harwell et al., 2012) and functional maturation of GABAergic inhibitory neurotransmission (Delmotte et al., 2020a,b). In the hippocampus, Shh is produced in both neurons and glial cells distributed in the pyramidal layer (Rivell et al., 2019; Tirou et al., 2020), and Shh receptors Ptch and Smo are further described to be expressed mostly at the postsynaptic membranes of CA1 and CA3 pyramidal neurons and at the mossy fibers terminals, thus indicating that the Shh-Smo pathway may regulate the strengthening of synaptic connectivity (Masdeu et al., 2007; Sasaki et al., 2010; Petralia et al., 2011; Mitchell et al., 2012).

Shh signaling can operate through canonical and non-canonical pathways (Carballo et al., 2018; Figure 2A). In the presence of ligand, Shh receptor Ptch release its constitutive inhibition on its signal transducer Smo receptor, leading to the activation of Shh canonical signaling effector (Carballo et al., 2018). Among canonical Shh signaling downstream targets are Smo and activator (Gli1 and Gli2^A^) or repressor (Gli3^R^) forms of Glioma-associated (Gli) gene transcription factors (Briscoe and Thérond, 2013; Rimkus et al., 2016; Carballo et al., 2018). In the central nervous system (CNS), during the late embryonic stage of mouse cortical development, canonical Shh-Smo signaling, acting through the inactivation of Gli3^R^, is essential to regulate the lineage change of cortical neural stem cells for the generation of olfactory bulb GABAergic interneurons (Zhang et al., 2020a). In hippocampal neurons, Shh is involved in axonal elongation through Smo and the canonical transcription factor Gli (Yao et al., 2015). Concurrently, several non-canonical, Gli-independent, Shh pathways have been described (Riobo et al., 2006; Ayers and Thérond, 2010). For instance, an interaction between Shh signaling and Ca^2+^-dependent spike activity in the developing spinal cord has been identified for spinal neuron differentiation and GABAergic phenotype homeostatic specification (Belgacem and Borodinsky, 2011; Belgacem et al., 2016). Moreover, this non-canonical Ca^2+^-dependent Shh signaling pathway has been shown to inhibit the canonical Shh signaling as spinal cord development progresses (Belgacem and Borodinsky, 2015).

## Non-Canonical Shh-Smo Signaling in Developing Gabaergic Networks

The developing hippocampus is characterized by spontaneous network oscillations called Giant Depolarizing Potentials (GDPs) that occur during the first postnatal weeks (Ben-Ari et al., 1989) and disappear when GABA currents became hyperpolarizing (Griguoli and Cherubini, 2017). In the developing human and mouse neocortex, Shh mRNA was identified in a subpopulation of glutamatergic and GABAergic neuronal cells (Komada et al., 2008; Memi et al., 2018). Interestingly, the gene expression profiles of both Ptch and Smo receptors in hippocampi of rat follow a similar pattern with a decreased expression levels when GABA became hyperpolarizing (Delmotte et al., 2020a). The GDPs could be modulated by several factors including neurotrophic factors such as brain-derived neurotrophic factor (BDNF) (Mohajerani et al., 2007), the metabolic hormone leptin (Dumon et al., 2019), the chemical stromal cell-derived factor-1-alpha (SDF-1; Kasyanov et al., 2006) and the hypothalamic neurohormone vasopressin (Spoljaric et al., 2017). More recently, Shh emerged as a new trophic factor in the functional maturation of GABAergic network (Delmotte et al., 2020a). *In vitro* experiments on hippocampal slices at postnatal days 5–7 showed that a non-canonical Ca^2+^-dependent Shh-Smo signaling increased the GDPs frequency and spontaneous GABAergic inhibitory postsynaptic currents without affecting glutamatergic synaptic activity (Delmotte et al., 2020a). The subcellular mechanism allowing the function of Shh-Smo pathway in GABAergic developmental plasticity depends on postsynaptic calcium-transients within the cytosol since the application of the Ca^2+^-chelator BAPTA in the recording pipette completely abolished this effect (Figure 2). This result suggests that Ca^2+^ serves as a second messenger in neuronal cells for the Smo receptor downstream pathway. However, the source for intracellular calcium signaling in neuronal cells following the binding of Shh or its agonist Shh Signaling Agonist (SAG; Chen et al., 2002) on their receptors remains to be determined (Figure 2). The interplay between Smo and Ca^2+^ activity has been also observed in embryonic spinal neurons where Smo receptor signaling leads to the recruitment of a heterotrimeric Gαi that in turn induces Inositol Triphosphate (IP3) oscillations apparent at the neuronal primary cilium synchronous with Ca^2+^ waves in the neuronal soma. The Shh-induced increase in Ca^2+^ spike activity depends on both intracellular Ca^2+^ stores and extracellular Ca^2+^ influx through voltage dependent calcium channels and transient receptor potential 1 channel (Belgacem and Borodinsky, 2011). Moreover, in embryonic spinal cord, the calcium increase generated by Smo signaling activates the PKA kinase which subsequently phosphorylates the cAMP response element-binding protein (CREB) transcription factor leading to the inhibition of Gli1 transcription factor (Belgacem and Borodinsky, 2015). These results are very similar to those observed during the first 2 weeks after birth in the rodent hippocampus where activation of the Smo signaling triggers phosphorylation of CREB as well as a decrease in Gli1 transcripts, suggesting an identical mechanism by a non-canonical Shh pathway (Delmotte et al., 2020a). Other Shh-Smo-mediated Ca^2+^ increases have been described under epileptic seizure discharges in hippocampal neurons through increased levels of extracellular glutamate and NMDA receptors activity (Feng et al., 2016). In this study, the authors suggest that Shh has no physiological function in adult hippocampal neuroplasticity. Interestingly, Delmotte et al. (2020a) reported that the application of SAG modulates the frequency of GDPs only during the first postnatal week of life and this action disappeared around P10. This divergence of the Shh-Smo effect between immature and mature hippocampal tissue may suggest that Shh-Smo signaling regulates the development of GABAergic neurotransmission only in immature neurons and disappears as soon as GABA becomes hyperpolarizing, thus reinforcing the hypothesis of a specific trophic role for Shh-Smo in the developing brain. This trophic function in the maturation of GABAergic neurotransmission acts through the regulated release of BDNF and activation of its high affinity tyrosine kinase receptor TrkB, suggesting that BDNF is a downstream target of Smo (Figure 1; Delmotte et al., 2020a). Other studies performed on different models of injury including cortical neurons, peripheral sciatic nerve, and cavernous nerve also showed an interplay between Shh-Smo and BDNF (Hashimoto et al., 2008; Dai et al., 2012; Bond et al., 2013). Although the subcellular mechanism remains to be demonstrated, a Shh-dependent increase of BDNF was found after SAG administration in lung organ cultures, and in hippocampal and spinal cord neuronal cells (Radzikinas et al., 2011; Liu et al., 2018; Delmotte et al., 2020a). In lung epithelial cells Shh promotes indirectly BDNF expression through a post-transcriptional mechanism, whereas in hippocampal neurons Shh-Smo triggered the dendritic release of BDNF *via* a calcium signal. These data indicate that Shh-Smo might acts through the BDNF–TrkB signaling pathway in both injured and healthy conditions.

## Canonical Shh-Smo Signaling Regulates Kcc2 Cell Surface Expression and Chloride Homeostasis

The postnatal maturation of GABAergic inhibitory transmission is linked to the developmental sequence of GABA shift from depolarizing to inhibitory actions which results from a developmental decrease in (Cl^−^)_i_, brought about by the increased contribution of the Cl^−^ extruder KCC2 (Rivera et al., 1999; Medina et al., 2014). Using molecular tools to manipulate Smo activity in the pyramidal neurons of the rat somatosensory cortex, Delmotte et al. (2020b) showed that Smo canonical signaling contributes to chloride ions homeostasis and KCC2 cell membrane stability (Delmotte et al., 2020b). Two Smo constructs were used, one mimicking Smo receptor in an activate state (Smo-CA) and a second blocking Smo signaling downstream pathway (Smo-ΔN). In this study, it has been demonstrated that Smo-CA accelerates the transition of GABA from depolarizing to hyperpolarizing, thus positioning the Shh-Smo pathway as a trigger of this GABAergic developmental sequence. This action on chloride ions homeostasis requires the activation of the Shh-Smo canonical signaling downstream target Gli1 as the inhibition of Gli1 abolished this effect (Delmotte et al., 2020b; Figure 1B). The abnormal GABAergic developmental sequence observed at the cellular level leads to behavioral consequences since electroporated rodents with the Smo-CA construct showed an increased susceptibility to seizures induced by the pentylenetrazol (PTZ), a chemical convulsive agent. In human, many studies illustrated compromised Cl^−^ homeostasis and altered GABAergic inhibition in patients with temporal lobe epilepsiy (TLE). On the other hand, an alteration of the Shh-Smo pathway has also been identified in TLE (Fang et al., 2011) and hypothalamic hamartoma with gelastic epilepsy (Hildebrand et al., 2016; Saitsu et al., 2016). Although the (Cl^−^)_i_ was not evaluated in the latter works, together above studies indicate the potential contribution of the Shh-Smo pathway to Cl^−^ disruption in TLE patients.

The key molecule controlling the resting level of neuronal Cl^−^ and directly linked to the switch of GABA from excitatory to inhibitory is KCC2 (Rivera et al., 1999; Virtanen et al., 2021 for recent comprehensive review). The dysfunctions of KCC2 are associated with large number of pathologies starting from pioneer observations of KCC2 change in different types of epilepsies (Miles et al., 2012) and ending with recent findings of KCC2 link to different neurological disorders (Tang, 2020). Naturally, the observations of Smo-dependent control on GABAergic polarity shift raised the question whether Smo transducer regulates the KCC2. The total expression level and the phosphorylated state of KCC2 are fundamental determinants of KCC2 function affecting its Cl^−^ extrusion ability and hence controlling neuronal Cl^−^ homeostasis (Box 1). Analysis of the phosphorylation state of Ser^940^ and Thr^1007^ residues of KCC2 revealed a strong and significant increase of Ser^940^ phosphorylation in neurons expressing Smo-CA, and significant decrease of Ser^940^ phosphorylation in neurons with Smo-ΔN (Delmotte et al., 2020b; Figure 2B). No effect of Smo was detected on the level of Thr1007 phosphorylation indicating on the high selectivity of Smo-dependent pathways controlling KCC2. Consistent with previous observations of Ser^940^ – dependent control of plasma membrane stabilization of KCC2 (Lee et al., 2007, 2011), the modulation of Smo pathway affected the surface expression of KCC2 construct harboring tag in extracellular domain and expressed in cultured hippocampal neurons (Delmotte et al., 2020b).

In the light of current knowledge on the regulation of KCC2 membrane trafficking, several hypotheses might be considered to explain the interplay between Smo and KCC2: (1) Activation of Smo downstream pathway with Smo-CA construct increases phosphorylation of KCC2 at Ser940 and consequently the stability of the co-transporter at the cell surface and its activity, thereby decreasing the intracellular chloride concentration and rendering GABA hyperpolarizing (Lee et al., 2007; Silayeva et al., 2015); (2) Complementary, the canonical Shh-Smo-Gli1 pathway may also regulate chloride ions homeostasis and KCC2 function through a second messenger including a Ca^2+^ transient frequency and/or an intermediary factor such as BDNF (Figure 1). As mentioned before, non-canonical Shh-Smo signaling pathway induce BDNF secretion and a previous study showed that the loss of canonical Shh-Smo signaling also decreased the levels of BDNF transcripts (Zhou et al., 2016). In line with this hypothesis, BDNF has been shown to modulate KCC2 expression and activity, but depending on the physiological context and maturation stage of neurons it can either upregulate (Ludwig et al., 2011) or down-regulate the KCC2 (Rivera et al., 2004); (3) A third hypothesis is based on the activation of Smo, which could affect the lipid organization of the membrane. Since the Smo binds sterols and endogenous cholesterol, a strong activation of the Smoothened receptor could prevent these bindings, leaving the unbound cholesterol free to move elsewhere in the membrane (Figure 1B). Cholesterol is an essential component of lipid rafts and is central to many mechanisms, including the stability of KCC2 at the membrane (Hartmann et al., 2009; Watanabe et al., 2009).

## Conclusion

In this review, we highlight the significant potential role of Shh-Smo signaling in chloride homeostasis through the control of KCC2 cell surface expression and activity, which in turn fine-tune the strength of inhibitory synaptic transmission. A weak or an absence of Shh-Smo signaling delayed the GABA polarity shift, leading to an unbalanced excitation to inhibition ratio. A similar role of Shh-smo signaling has been shown on astrocytes in mature cortex (Hill et al., 2019). The conditional knockout of Smo results in an early increase in synapse number and in neuronal excitability, induced by a reduction in Kir 1.4 potassium inward rectifier currents, thus confirming an important role for the Shh pathway on synaptic transmission. It remains to determine whether modulation of Ptch receptor with Shh will have the same consequences as Smo activation on GABAergic developmental sequence. Indeed, activation of Smo by SAG or stimulation of Ptch with application of Shh results in the same outcome in a very large number of functions, both for regulation of axonal elongation and, activation of the ERK signaling pathway (Yao et al., 2015, 2019). Finally, these recent data on Shh-Smo showed that this pathway continues to have a very important role in development after birth, both in early network activity such as GDPs and in the maturation of inhibitory synaptic transmission with the developmental GABAergic sequence. This signaling pathway can trigger the release of a key neurotrophic factor in CNS development, BDNF, but also modulate the activity of a central protein in chloride homeostasis and GABAergic polarity control, KCC2.

These open new research avenues on the interplay between Shh and GABA in neurodevelopmental disorders like Autism Spectrum Disorders (ASD), Down Syndrome and, epilepsy (Belgacem et al., 2016; Feng et al., 2016; Kumar et al., 2019). As promising examples, treatments with SAG restored partially cerebellar morphology and behavioral deficits in a Down Syndrome mouse model (Roper et al., 2006; Das et al., 2013), and purmorphamine, a Smo agonist, ameliorated behavioral and cellular alterations in a rat model of ASD (Rahi et al., 2021).

BOX 1: Phosphorylation-dependent control of KCC2.

A large number of phosphorylatable residues were revealed on KCC2 using mass spectrometry analysis (Rinehart et al., 2011; Weber et al., 2014; Agez et al., 2017; Cordshagen et al., 2018; Zhang et al., 2020b). The mutation of some residues (e.g., Ser^932^, Thr^934^, Ser^937^, Ser^940^, Thr^906^, Thr^1007^) results in change of the ion-transport activity of KCC2 measured in heterologous expression system (Weber et al., 2014; Zhang et al., 2020b). The importance of other residues is not clear yet. Among above phosphorylatable sites with ion-transport importance only two group of residues (Ser^940^ and Thr^906^ plus Thr^1007^) were extensively studied for their role in native neuronal environment both *in-vivo* and *in-vitro*. Briefly, the protein kinase C (pkC) phosphorylation of Ser^940^ leads to stabilization of KCC2 on neuron’s surface leading to enhancement of ion-transport activity, whereas Ser^940^ dephosphorylation is associated with transporter internalization and reduction of the KCC2 activity (Lee et al., 2007, 2011). The transgenic mice harboring nonphosphorylatable mutation S940A showed reduced sociability that is compatible with autism-like behavior (Moore et al., 2019) and exhibited higher lethality when entering in kainate-induced status epilepticus (Silayeva et al., 2015). The phosphorylation of Thr^906^ and Thr^1007^ is associated with decrease of ion-transport ability of KCC2 (Rinehart et al., 2009; Friedel et al., 2015) due to enhancement of KCC2’s internalization rate (Friedel et al., 2015). In opposite, the dephosphorylation of Thr^906^ and Thr^1007^ results in increase of KCC2 activity and enhancement of its surface expression (Friedel et al., 2015). The mouse harboring phosphomimetic T906E/T1007E mutations in both alleles dies at birth (Watanabe et al., 2019). The heterozygous T906E/T1007E mouse is characterized by deficit in neuronal Cl^−^ homeostasis, network activity and has altered social interaction behavior (Pisella et al., 2019). In opposite, the mouse with T906A/T1007A mutations, mimicking non-phosphorylated state of respective threonine residues, exhibited increased basal neuronal Cl − extrusion, showed delayed onset and severity of chemoconvulsant-induced seizure activity (Moore et al., 2018), and exhibited enhanced social behavior (Moore et al., 2019). The phosphorylation of both Thr^906^ and Thr^1007^ depend on activity of with no lysine kinase (WNK) including four members (WNK1–4) and the downstream SPAK/OSR1 (SPS1-related proline/alanine-rich kinase/oxidative stress responsive kinase-1) pathway (Alessi et al., 2014; Friedel et al., 2015), although the exact mechanisms of KCC2 phosphorylation by WNK/SPAK remain to be clarified.

## Author Contributions

The review was conceptualized, written, and edited by each of the authors. CP was the supervisor. All authors contributed to the article and approved the submitted version.

## Funding

This work was supported by The National Institute of Health and Medical Research (INSERM), the National Center for Scientific Research (CNRS), the A*MIDEX project (ANR-11-IDEX-0001-02) funded by the “Investissements d’Avenir” French Government Program, managed by the Agence Nationale de la Recherche. IM was supported by French Foundation for Epilepsy Research. MH was supported by a Doctoral fellowship from the French Ministry of higher Education and Research.

## Conflict of Interest

The authors declare that the research was conducted in the absence of any commercial or financial relationships that could be construed as a potential conflict of interest.

## Publisher’s Note

All claims expressed in this article are solely those of the authors and do not necessarily represent those of their affiliated organizations, or those of the publisher, the editors and the reviewers. Any product that may be evaluated in this article, or claim that may be made by its manufacturer, is not guaranteed or endorsed by the publisher.
